# Supplementary figures and images for: Transplanted Murine Tumours SPECT Imaging with 99mTc Delivered with an Artificial Recombinant Protein
Source: Int J Mol Sci. 2024 Sep 23;25(18):10197. doi: 10.3390/ijms251810197 (PMC11432708; doi:10.3390/ijms251810197)

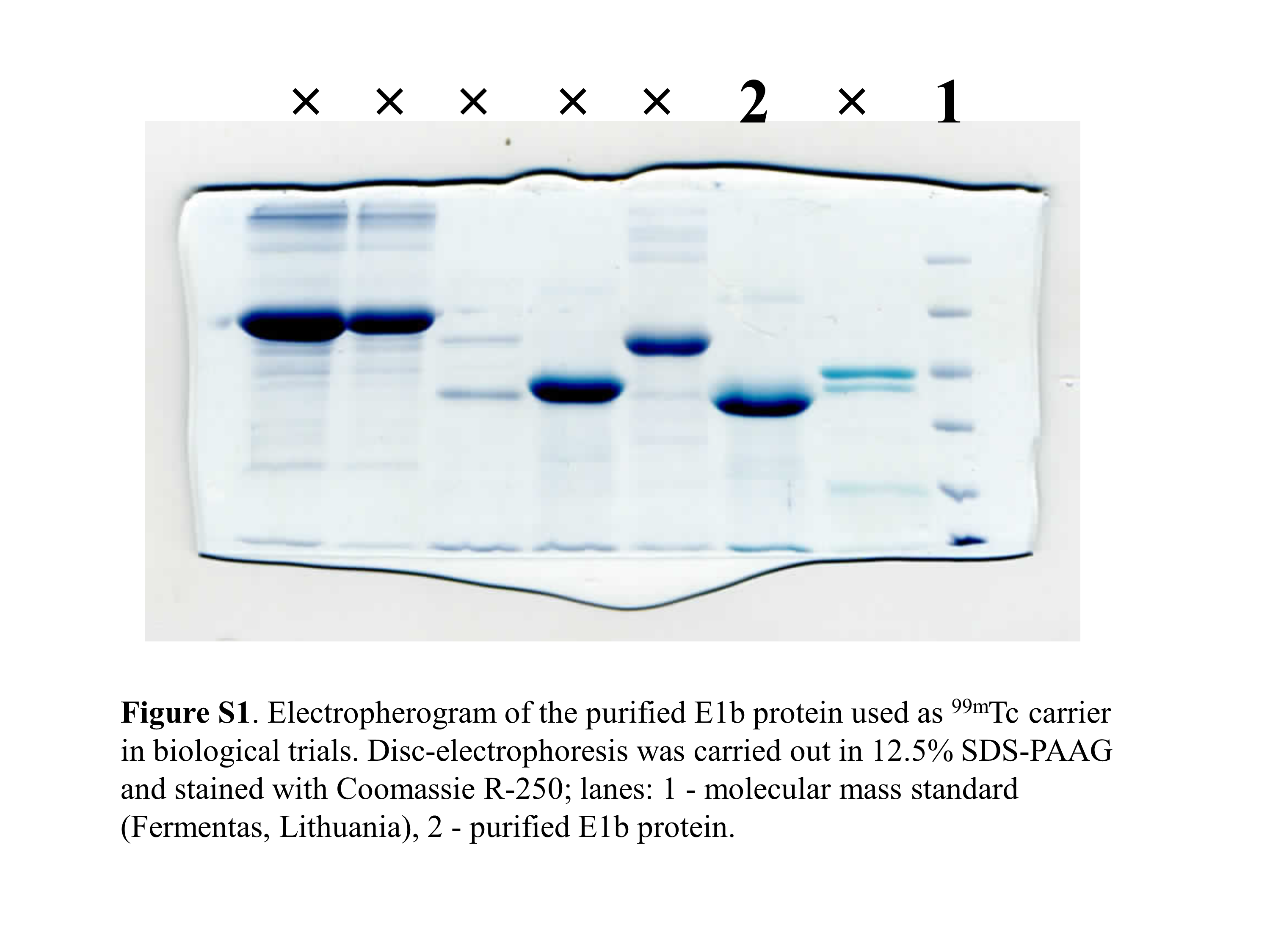

Supplement: Supplementary file 1 [file ijms-25-10197-s001.zip › Supporting information/Fugure S1_Electropherogram of the purified E1b protein.tif]
